# Study on the effects of combined tea drinking and mental activity after dinner on smokers in China

**DOI:** 10.18332/tid/150654

**Published:** 2022-07-29

**Authors:** Zhongbo Chen, Xuechan Yu, Hanlu Gao, Jie Cen, Qianqian Xu, Jing Gong, Sha Li, Mianzhi Ye, Dan Lv, Hui Chen, Hongying Ma, Younuo Wang, Qingwen Su, Yiming Yu, Zaichun Deng

**Affiliations:** 1 Department of Pulmonary and Critical Care Medicine, The Affiliated Hospital of Medical School, Ningbo University, Ningbo, China; 2 Department of Prevention and Health Care, The Affiliated Hospital of Medical School, Ningbo University, Ningbo, China; 3 Department of Pulmonary and Critical Care Medicine, Ningbo Ninth Hospital, Ningbo, China; 4 Ningbo Municipal Center for Disease Control and Prevention, Ningbo, China; 5 The Center for Disease Control and Prevention of Jiangbei District, Ningbo, China

**Keywords:** cigarettes, nicotine dependence, tea, mental activities after dinner, smoke

## Abstract

**INTRODUCTION:**

Cigarette and tobacco use is a leading cause of chronic obstructive pulmonary disease, lung cancer, and other malignant tumors. In China, people prefer to engage in mental activities (gambling, overtime work, playing video games, or other mental activities) on the weekends or during spare time, especially in the evening before they prepare for bed. In China, smokers frequently consume tea while smoking. The relationship between smokers who consume tea, engage in mental activities after dinner, or both (drinking tea and engaging in cognitive activities after dinner together), and daily cigarette smoking or nicotine addiction must be clarified.

**METHODS:**

A total of 438 smokers were included in the study. Age, gender, body mass index (BMI), smoking habits, Fagerström test for nicotine dependence scores, and behaviors, were recorded. The study excluded smokers with a Fagerström score <1 or with a mental disorder diagnosis. The smokers were divided into four groups based on their behaviors: those who did not drink tea, did not engage in mental activities after dinner, those who drank tea only, those who engaged in mental activities only, and those who engaged in both.

**RESULTS:**

Only drinking tea or doing mental activities after dinner cannot increase cigarettes per day (22.20 ± 10.143 vs 23.49 ± 11.966, p=0.362; 22.20 ± 10.143 vs 22.66 ± 1.192, p=0.750) or FTND scores [6.0 (4.0; 7.0) vs 6.0 (4.0; 7.75), p=0.941; 6.0 (4.0; 7.0) vs 6.0 (4.25; 7.75), p=0.980]. People who drink tea and engage in mental activities after dinner smoke more (22.20 ± 10.143 vs 30.75 ± 17.264, p<0.0001) and have higher nicotine dependence levels [6.0 (4.0; 7.0) vs 7.0 (5.0; 8.0), p=0.015].

**CONCLUSIONS:**

The consumption of tea or a mental activity after dinner is not associated with daily smoking or nicotine dependence. There is an association between the combined behaviors (tea drinking and mental activity after dinner) and the daily consumption of cigarettes, and the degree of nicotine dependence.

## INTRODUCTION

Consuming cigarettes or smoking is a common factor that leads to chronic obstructive pulmonary disease^1^, lung cancer^2^, and other malignant tumor diseases^3^. Nicotine dependence has been defined as a mental disorder^4^ with withdrawal symptoms like other drugs. There are many behavioral factors involved in smoking, and each smoker has a different lifestyle, and hence smoking can occur across a variety of environments. We have summarized a unique environment from previous literature that explains clearly why smoking and behaviors coexist: the progression of mental activities. It has the same properties that increase dopamine release and dopamine consumption^5,6^. Ex-smokers or never-smokers who work overtime are more likely to become smokers because of increased stress and exhaustion^7,8^. Gambling and playing video games are two environmental motivational stimuli which, combined with smoking or other addictive substances, may increase dopamine in the nucleus accumbens^6,9,10^. It is customary in China for people to attend mental activities on weekends or during the evening hours after dinner. However, few studies have explained the relationship between mental activities after dinner, which can stimulate the mind, and daily consumption of cigarettes as well as nicotine dependence.

Chinese smokers like drinking tea while working or engaging in mental activities. The main component of tea is caffeine, but it is not completely consistent with the caffeine component in coffee beverages^11^. The relationship between smoking and drinking tea has not been explained specifically in previous research^12,13^.

Furthermore, there is limited research comparing the number of cigarettes smoked and nicotine dependence with smokers’ habits of drinking tea and engaging in mental activities after dinner. Therefore, we collected smokers’ information and behaviors from clinic records. To clarify whether smokers with drinking tea, mental activities after dinner or these two behaviors in combination, consume more cigarettes or are more nicotine dependent, the daily cigarettes consumed and the Fagerström scores were used as indexes of smoking severity.

## METHODS

### Patients and information collection

From June 2009 to August 2020, 438 smokers were retrospectively enrolled at The Affiliated Hospital of Medical School, Ningbo University. As the smokers came to our clinic for counseling and treatment, the doctor recorded their habits and all other information. Our study collected and analyzed these data, retrospectively. The study was approved by the Ethics Committee of the Affiliated Hospital of Medical School, Ningbo University (Ningbo, China). The methods used were in accordance with NCCN Clinical Practice Guidelines in Oncology of Smoking Cessation^14^.

### Study design

Age, gender, Body Mass Index (BMI, kg/m^2^), and the number of cigarettes consumed per day were recorded. Smokers with FTND <1 or with a diagnosis of a mental disorder were excluded from the study. In addition, the FTND scores were used to evaluate the level of nicotine dependence^15,16^. The following behaviors were observed: 1) drinking tea; and 2) participating in mental activities after dinner before sleeping for at least one hour (including gambling^10^, overtime work^7^, and playing video games^17^).

Smokers who smoked during the period of mental activities after dinner before sleep were included in the study. Since the plasma half-life of nicotine is 2 hours^18^, we did not record the number and duration of cigarettes smoked. Among patients with tobacco addiction, at least three of the following six criteria must be met^19-21^: 1) craving, or a strong desire or urge to use tobacco; 2) persistent desire or unsuccessful efforts to cut down or control tobacco use; 3) experiencing tobacco withdrawal symptoms (such as irritability, frustration, anger, anxiety, difficulty concentrating, increased appetite, restlessness, insomnia) after abrupt cessation of tobacco use, or reduction in the amount of tobacco used; 4) tolerance, defined as the need for markedly increased amounts of tobacco to achieve the desired effect; 5) given up or reduced important social, occupational, or recreational activities because of tobacco use; and 6) continued tobacco use, despite knowledge of having a persistent or recurrent physical or psychological problem that is likely to have been caused or exacerbated by tobacco. We firstly excluded patients without all these conditions because they were deemed to be not addicted to tobacco. None of the patients with FTND <1 met these conditions.

Smokers were divided into four groups according to behaviors: 1) no drinking tea and no mental activities after dinner group; 2) only drinking tea; 3) only mental activities after dinner; and 4) combination behaviors (drinking tea and mental activities after dinner together).

### Statistical analysis

Fisher’s exact test was used to calculate the gender distribution. Mean ± standard deviation (SD) and t-test were used for age, BMI and cigarette consumption. FTND scores are presented as median (Q1, Q3) and were calculated by Mann-Whitney U test. Cigarette consumption and FTND score of tea group, mental activities after dinner group, combination behaviors group, were all compared with no tea no mental activities group, respectively. IBM SPSS Statistics 21.0 was used for all statistical analyses. A value of p<0.05 was judged as statistical different. There is no comparison between the groups, so the alpha value does not need to be corrected.

## RESULTS

The age, BMI, and gender distribution of smokers in four groups were not significantly different. Table 1 indicates that only drinking tea or engaging in mental activities after dinner was not related with the number of cigarettes per day (22.20 ± 10.143 vs 23.49 ± 11.966, p=0.362; 22.20 ± 10.143 vs 22.66 ± 1.192, p=0.750) or FTND scores [6.0 (4.0; 7.0) vs 6.0 (4.0; 7.75), p=0.941; 6.0 (4.0; 7.0) vs 6.0 (4.25; 7.75), p=0.980]. Patients who combine drinking tea with mental activities after dinner were found to smoke more (22.20 ± 10.143 vs 30.75 ± 17.264, p<0.0001) and display higher levels of nicotine dependence [6.0 (4.0; 7.0) vs 7.0 (5.0; 8.0), p=0.015].

**Table 1:** Cigarette consumption and nicotine dependence in different groups

*Characteristics*	*No drinking tea or mental activities after dinner (n=124)*	*Only drinking tea (n=124)*	*p*	*Only mental activities after dinner (n=97)*	*p*	*Drinking tea and mental activities after dinner (n=93)*	*p*
**Age** (years), mean ± SD	43.54 ± 12.142	47.39 ± 10.336	0.008	39.72 ± 10.925	0.016	43.45 ± 11.222	0.089
**Gender**, n							
Male	114	115	1	88	0.811	90	0.16
Female	10	9		9		3	
**BMI** (kg/m²), mean ± SD	24.183 ± 9.291	23.697 ± 2.827	0.578	23.019 ± 3.093	0.238	22.708 ± 3.852	0.151
**Daily cigarette consumption**, mean ± SD	22.20 ± 10.143	23.49 ± 11.966	0.361	22.66 ± 1.192	0.75	30.75 ± 17.264	<0.0001*
**FTND score**, median (Q1, Q3)	6.0 (4.0; 7.0)	6.0 (4.0; 7.75)	0.941	6.0 (4.25; 7.0)	0.98	7.0 (5.0; 8.0)	0.015*

* p<0.05 was considered as statistical difference. Because there is no comparison between groups, there is no need to correct the alpha value. Cigarette consumption was calculated by t-test analysis in tea group, mental activities after dinner group, tea and mental activities after dinner group. All compared with no tea no mental activities group, respectively. FTND score was calculated by Mann-Whitney U test in tea group, mental activities after dinner group, tea and mental activities after dinner group. All compared with no tea no mental activities group, respectively.

## DISCUSSION

Several malignant diseases are associated with smoking^3^, and smoking is an increasingly significant public health issue worldwide. Smokers often engage in mental activities after dinner in China, where it is common to drink tea while smoking^22^. According to the characteristics of smokers in smoking cessation clinics, mental activities and drinking tea after dinner are long-term behaviors and cumulative processes. We cannot track these activities in a prospective manner. The majority of people do not experience these factors at the same time. There was a statistically significant difference in the number of cigarettes smoked per day among people who did not engage in mental activities after eating dinner and did not consume caffeinated beverages, based on previous research^13,23,24^. On the basis of these findings, we set out to investigate the effect of drinking tea and engaging in mental activity after dinner on smoking. In our study, smokers with combined behaviors (drinking tea and participating in mental activities after dinner) smoked more and had higher nicotine dependence scores than smokers drinking tea or participating in mental activities alone.

Chinese smokers prefer green tea, and caffeine’s constituents function in tea differently from those in coffee. Green tea requires a long infusion period since Chinese smokers prefer to steep leaves continuously in cups by adding boiling water repeatedly until the leaves no longer have a taste. In this way, the caffeine contained in green tea is fully released into the water. Caffeine consumption is higher in Chinese smokers^22^. The main constituents of tea are polyphenols, catechins, theanine, proteins, enzymes, caffeine, carbohydrates, and inorganic compounds^11^. Despite the fact that cups of coffee generally contain 1.4 to 3.4 times more caffeine than cups of tea^25^, drinking tea every day provides a chronic caffeine exposure environment conducive to shaping and maintaining tobacco addiction^24^. Additionally, the caffeine in tea has a faster response time than theanine^26^, which explains why some smokers continue to crave cigarettes after drinking tea. Drinking caffeine beverages increases anxiety levels^27,28^ so smokers use nicotine to relieve anxiety by providing a calming effect^28,29^.

However, drinking tea or caffeine beverages cannot take full responsibility for the number of cigarettes smoked per day^13^. Although smokers drink more coffee than non-smokers or ex-smokers, not every smoker drinks coffee, or tea, and smokes at the same time^13,24^. The behavior of engaging in mental activities after dinner, which increases dopamine consumption in smokers, increases the risk of smoking more and has been associated with a higher FTND score^7,10,17,30,31^. Yet, no research has been able to demonstrate an increase in cigarettes or FTND scores in these smokers. According to our data, patients in only the mental activities group smoke the same number of cigarettes per day. It is possible that the results are due to the choice of smokers as the control group (no tea, no mental activities) rather than non-smokers or ex-smokers. As a results, drinking only tea will not increase cigarette consumption or FTND level, implying the same conclusion from data.

The data critically indicate that smokers with behaviors such as drinking tea and engaging in mental activities after dinner smoke more and score higher on the FTND scale. On the one hand, Chinese often work during their spare time or on the weekends, especially after dinner and during the evening. Smokers who work overtime may be stressed^7^. Stress is the third variable when smokers drink coffee during mental activities^8,32^. Perceived stress has been strongly associated with nicotine withdrawal symptomatology and may exacerbate nicotine withdrawal symptoms^33^. Conditions, caffeine intake, or the combination of stress and condition, contribute to the increase in the number of cigarettes consumed^13^. Alternatively, mental activities after dinner may occur in conjunction with smoking and drinking tea. There are two reasons for this. First, a brief outline of the role of dopamine and its deficiencies is as follows: dopamine helps to get rid of tiredness after all day work^34^; dopamine also helps focus attention if the brain is not well rested^34^; and dopamine deficiency occurs during mental activities due to inverted-U-shaped (dopamine was consumed by the brain) dopamine actions on memory and cognitive control^5^, especially in drug addicts and smokers^35^. Second, are the choices of dopamine supplementation during mental activities, as tea is available as easy as coffee in China; the sedative effect of dopamine produced by nicotine would relieve the anxiety^24^ and stress caused by the caffeine found in tea^24,28^ and the mental activities. Furthermore, the co-production of dopamine by nicotine and caffeine at the same time could help focus greater attention during mental activities^24^. As a result, smokers who consume tea and engage in mental activities after dinner may have a higher level of nicotine dependence and more cigarettes consumed per day.

### Limitations

There are some limitations to our research. First, we were unable to compile a detailed record of mental activities along with an assessment of patient stress and anxiety, and hence we were unable to explore more additional traits. Second, further information on the type of tea, amount of tea consumed, and the manner in which smokers in China drink tea was not recorded. Third, lifestyle behaviors may have interacted effects with each other, which need to be explained more specifically in future studies such as whether smoking would lead to drinking tea. Fourth, the information we collected was from smokers who attended smoking cessation clinics, and all of them had obvious signs of physical addiction to smoking. However, the proportion of people quitting smoking in China remains low, and there is not much awareness of the benefits of quitting^36^. There are many environmental factors that contribute to increased smoking, but some of these factors are psychological rather than physical. Although there are many factors that contribute to smoking, our study is only a preliminary summary of these patient characteristics. Further research is needed with studies of more appropriate design.

## CONCLUSIONS

Drinking tea or engaging in mental activity after dinner was not related with higher nicotine dependence in smokers; however, smokers with combined behaviors (consume tea and engage in mental activities after dinner) have higher cigarette consumption and nicotine dependence levels.

## Data Availability

The data supporting this research are available from the authors on reasonable request.

## References

[1] Willemse BW, Postma DS, Timens W, ten Hacken NH (2004). The impact of smoking cessation on respiratory symptoms, lung function, airway hyperresponsiveness and inflammation. Eur Respir J.

[2] John A, Yang B, Shah R (2021). Clinical Impact of Adherence to NCCN Guidelines for Biomarker Testing and FirstLine Treatment in Advanced Non-Small Cell Lung Cancer (aNSCLC) Using Real-World Electronic Health Record Data. Adv Ther.

[3] Siegel RL, Miller KD, Fuchs HE, Jemal A (2021). Cancer Statistics, 2021. CA Cancer J Clin.

[4] Robins LN, Helzer JE, Croughan J, Ratcliff KS (1981). National Institute of Mental Health Diagnostic Interview Schedule. Its history, characteristics, and validity. Arch Gen Psychiatry.

[5] Cools R, D'Esposito M (2011). Inverted-U-shaped dopamine actions on human working memory and cognitive control. Biol Psychiatry.

[6] Chambers RA, Taylor JR, Potenza MN (2003). Developmental neurocircuitry of motivation in adolescence: a critical period of addiction vulnerability. Am J Psychiatry.

[7] Angrave D, Charlwood A, Wooden M (2014). Working time and cigarette smoking: evidence from Australia and the United Kingdom. Soc Sci Med.

[8] Finlay JM, Zigmond MJ (1997). The effects of stress on central dopaminergic neurons: possible clinical implications. Neurochem Res.

[9] Koepp MJ, Gunn RN, Lawrence AD (1998). Evidence for striatal dopamine release during a video game. Nature.

[10] Grant JE, Potenza MN (2005). Tobacco use and pathological gambling. Ann Clin Psychiatry.

[11] Mohanpuria P, Kumar V, Yadav SK (2010). Tea caffeine: Metabolism, functions, and reduction strategies. Food Sci Biotechnol.

[12] Prineas RJ, Jacobs DR, Crow RS, Blackburn H (1980). Coffee, tea and VPB. J Chronic Dis.

[13] Swanson JA, Lee JW, Hopp JW (1994). Caffeine and nicotine: a review of their joint use and possible interactive effects in tobacco withdrawal. Addict Behav.

[14] Shields PG, Herbst RS, Arenberg D (2016). Smoking Cessation, Version 1.2016, NCCN Clinical Practice Guidelines in Oncology. J Natl Compr Canc Netw.

[15] Payne TJ, Smith PO, McCracken LM, McSherry WC, Antony MM (1994). Assessing nicotine dependence: a comparison of the Fagerström Tolerance Questionnaire (FTQ) with the Fagerström Test for Nicotine Dependence (FTND) in a clinical sample. Addict Behav.

[16] Ray R, Schnoll RA, Lerman C (2009). Nicotine dependence: biology, behavior, and treatment. Annu Rev Med.

[17] Raiff BR, Jarvis BP, Rapoza D (2012). Prevalence of video game use, cigarette smoking, and acceptability of a video gamebased smoking cessation intervention among online adults. Nicotine Tob Res.

[18] Hukkanen J, Jacob P, Benowitz NL (2005). Metabolism and disposition kinetics of nicotine. Pharmacol Rev.

[19] World Health Organization (1992). The ICD-10 Classification of Mental and Behavioral Disorders: Clinical descriptions and diagnostic guidelines.

[20] China National Health and Family Planning Commission Statement of China National Health and Family Planning Commission of China.

[21] American Psychiatric Association Diagnostic and statistical manual of mental disorders DSM-5.

[22] Astill C, Birch MR, Dacombe C, Humphrey PG, Martin PT (2001). Factors affecting the caffeine and polyphenol contents of black and green tea infusions. J Agric Food Chem.

[23] Yu X, Yu Y, Ma H, Chen Z, Deng Z (2021). Mental activities after dinner increase cigarettes consumption. Sci Rep.

[24] Tanda G, Goldberg SR (2000). Alteration of the behavioral effects of nicotine by chronic caffeine exposure. Pharmacol Biochem Behav.

[25] Food Standards Agency (2004). Survey of caffeine levels in hot beverages. Food Survey Information Sheets.

[26] Rogers PJ, Smith JE, Heatherley SV, PleydellPearce CW (2008). Time for tea: mood, blood pressure and cognitive performance effects of caffeine and theanine administered alone and together. Psychopharmacology.

[27] Chou TM, Benowitz NL (1994). Caffeine and coffee: effects on health and cardiovascular disease. Comp Biochem Physiol C Pharmacol Toxicol Endocrinol.

[28] Vinader-Caerols C, Monleón S, Carrasco C, Parra A (2012). Effects of Alcohol, Coffee, and Tobacco, Alone or in Combination, on Physiological Parameters and Anxiety in a Young Population. J Caffeine Res.

[29] Gilbert DG (1979). Paradoxical tranquilizing and emotionreducing effects of nicotine. Psychol Bull.

[30] Chen A, Machiorlatti M, Krebs NM, Muscat JE (2019). Socioeconomic differences in nicotine exposure and dependence in adult daily smokers. BMC Public Health.

[31] McGrath DS, Barrett SP (2009). The comorbidity of tobacco smoking and gambling: a review of the literature. Drug Alcohol Rev.

[32] Self DW, Nestler EJ (1998). Relapse to drug-seeking: neural and molecular mechanisms. Drug Alcohol Depend.

[33] Kassel JD, Stroud LR, Paronis CA (2003). Smoking, stress, and negative affect: correlation, causation, and context across stages of smoking. Psychol Bull.

[34] Gray JA, Young AM, Joseph MH (1997). Dopamine's role. Science.

[35] Nutt DJ, Lingford-Hughes A, Erritzoe D, Stokes PRA (2015). The dopamine theory of addiction: 40 years of highs and lows. Nat Rev Neurosci.

[36] Zhang M, Wang LM, Li YC (2012). Cross-sectional survey on smoking and smoking cessation behaviors among Chinese adults in 2010. In Chinese. Zhonghua Yu Fang Yi Xue Za Zhi.

